# [Retracted] Downregulation of miR‑29b targets DNMT3b to suppress cellular apoptosis and enhance proliferation in pancreatic cancer

**DOI:** 10.3892/mmr.2023.13049

**Published:** 2023-07-10

**Authors:** Li-Hua Wang, Ju Huang, Cheng-Rong Wu, Liu-Ye Huang, Jun Cui, Zhi-Zhi Xing, Chun-Yu Zhao

Mol Med Rep 17: 2113–2120, 2018; DOI: 10.3892/mmr.2017.8145

Following the publication of this paper, it was drawn to the Editor's attention by a concerned reader that certain of the Hoechst staining data shown in Fig. 4E were strikingly similar to data appearing in different form in another article by different authors at a different research institute; moreover, an unexpectedly high degree of similarity was noted with the data featured in a couple of different data panels showing the results of apoptosis experiments in Fig. 4D. Owing to the fact that the contentious data in the above article had already been published prior to its submission to *Molecular Medicine Reports*, the Editor has decided that this paper should be retracted from the Journal. The authors were asked for an explanation to account for these concerns, but the Editorial Office did not receive a reply. The Editor apologizes to the readership for any inconvenience caused.

